# Evaluation of Single-Event Effect Current-Carrier Mapping Based on Experimental Data

**DOI:** 10.3390/mi15111353

**Published:** 2024-11-06

**Authors:** Mengtian Bao, Ying Wang, Jianqun Yang, Xingji Li

**Affiliations:** 1 School of Information Science and Technology, Dalian Maritime University, Dalian 116026, China; baomengtian@163.com; 2 National Key Laboratory of Materials Behavior and Evaluation Technology in Space Environment, Harbin Institute of Technology, Harbin 150080, China; yangjianqun@hit.edu.cn (J.Y.); lxj0218@hit.edu.cn (X.L.)

**Keywords:** data analysis, single-event effect, power MOSFET, evaluation

## Abstract

For single-event radiation damage of power MOSFET devices, this paper aims to establish a statistical analysis method based on external observation (gate/drain current characteristics in irradiation environment) to recognize and evaluate the radiation evolution process and damage mechanism of microscopic physical quantities inside the devices, namely current-carrier (CC) mapping. Firstly, a special data fluctuate–collapse transform analysis method is proposed according to the temporal characteristics of the gate/drain current. Secondly, a carrier dynamic balance ratio based on current data is defined to evaluate the radiation damage degree of the device. TCAD is used to deeply study the relationship between the external current characteristics and the evolution process of internal physical quantities and the damage mechanism. The results show that the current data timing analysis based on fluctuate–collapse transformation can better peer into the evolution process of irradiation events inside the device, and the statistical analysis based on the dynamic balance ratio of carriers can evaluate the severity of irradiation damage to a certain extent.

## 1. Introduction

Power metal–oxide–silicon field-effect transistor (MOSFET) devices are widely used as core components of power electronic systems in aerospace, electric vehicles, high voltage power transmission, and other fields [1,2,3,4]. When power MOSFET devices are applied in a cosmic environment, the impact of the single-event effect produced by heavy ions present in space on device performance needs to be considered [5,6,7,8]. Unlike the single-event transient (SET) effect caused by heavy-ion incident nano-devices on structures and application circuits, which leads to soft errors in logic circuits [9,10], for power MOSFET devices, heavy ions are susceptible to another two of the most serious effects, the Single-Event Burnout (SEB) effect and the Single-Event Gate Rupture (SEGR) effect [11,12,13]. These two effects affect the device leakage current and increase the electric field in the gate dielectric layer, leading to degradation of device performance and, in severe cases, destructive damage.

At present, the research on the single-event effect of power MOSFET devices mainly focuses on damage mechanism analysis and reinforcement structure design [14,15,16,17,18,19]. The above studies focus on the cause of damage caused by single-event effects on the device from a microscopic perspective, and there are few macro-evaluation studies on single-event effects in power MOSFET devices [20]. Some researchers carried out the Singel-Event Upset (SEU) rate prediction model based on the ion incident path length and LET value [21,22]. Subsequently, a semi-empirical model of the proton SEU rate was established based on experiments. On this basis, corresponding codes and software were developed. The prediction of the single-event effect in this period was centered around the SEU in the circuit, and the above prediction model was not applicable to the power MOSFET devices. On the other hand, some scholars carried out research on the prediction of early SEGR failure of VDMOS in commercial space systems, discussed the concept of lethal ion rate, and utilized the extracted expressions, integral flux curves, and other means to carry out the early failure prediction [23]. The above prediction methods provide assistance in the selection of parameters such as vehicle orbit conditions, shield thickness, and so on. After 2000, most of the prediction studies on single-event effects focused on the organization and comparison of past evaluation models [24]. Previous single-event effect evaluation studies have focused on the SEU rate and simulation procedures, and there is a lack of in-depth research on the changes in the timing patterns of the gate and drain currents in the irradiation experiments and the reasons for their formation. The existing theoretical analysis of the device voltage and structure has limitations.

The background of this research is the experimental evaluation of the irradiation effects of Si-power MOSFET devices studied by the collaborative team. Under the background of theoretical research on the triggering mechanism of the radiation effect and the principle of radiation damage, our interest lies in the processing and analysis of radiation experimental data, hoping to obtain statistical observation methods that are consistent with the above-related theories. Therefore, this paper introduces a data analysis method to study single-event effects in power MOSFET devices, breaking through the limitations of device voltage withstand and structure on theoretical analysis and establishing a mapping mechanism from external macroscopic currents to internal microscopic carriers, i.e., the current-charge mapping principle (CC mapping), after irradiation. While investigating the test results and triggering mechanism of single-event effects in power devices, the evaluating method for the internal damage of devices by external currents is provided. Supplement the experience and theoretical cognition of single-event radiation damage of power semiconductor devices are hoped through the research in this paper. Under the condition that experimental resources are limited and only irradiation experiments can be carried out, the work in this paper is helpful in judging the characteristics and rules of carrier motion changes in the device during irradiation based on experimental data, and further research is expected to establish an AI evaluation model of irradiation damage by single-event effect.

## 2. Experimental Introduction and Result

### 2.1. Samples and Experimental Platforms

The single-event effect experiments were conducted with a Ta ion radiation beam at the Institute of Modern Physics, Chinese Academy of Sciences (Lanzhou, China). ^181^Ta^35+^ ion beam with the energy of 1912.1 MeV, the range of 111.3 μm (Si), and the LET of 76.3 MeV·cm^2^/mg was incident vertically on the samples. A total of 55 valid samples were obtained from 8 models of the N-channel Si MOSFET sample devices. To facilitate subsequent data analysis, the above 8 models of devices and the number of valid samples in each type of device are shown in Table 1. The breakdown voltages (*BV*) of the sample devices are, respectively, and the subscripts show the type of different samples: *BV*_I_ = *BV*_VIII_ = 650 V, *BV*_II_ = *BV*_VI_ = 150 V, *BV*_III_ = *BV*_V_ = 800 V, *BV*_IV_ = 40 V, *BV*_VII_ = 1500 V. Information about the different types of samples is described below.

### 2.2. Experimental Result

To ensure that the tested sample is fully irradiated and the tested sample is completely damaged, this paper determined that the condition for the tested sample to occur SEB or SEGR is that the test system has drain overcurrent protection or gate overcurrent protection, that is, the continuous sampling value of the drain current or gate current exceeds 100 mA. In the single-event irradiation test, when the SEB or SEGR effect occurs in the tested sample or the fluence amount exceeds 10^6^/cm^2^, the irradiation experiment is terminated. In this paper, the above three criteria are described as the interruption mode of a single-event irradiation experiment, namely drain overcurrent, gate overcurrent, and fluence overlimit. Considering the irradiation duration, the probability of occurrence of the above three interruption modes in 55 samples is shown in Table 2.

(1) Current timing pattern

In theory, the temporal responses of the *I*_GS_ and *I*_DS_ before beam supply are both stationary random processes. After heavy-ion irradiation, the temporal response of the *I*_GS_ and *I*_DS_ is usually a non-stationary stochastic process. Based on the gate and drain current data of the samples, we classify the timing patterns of current changes into the following four categories: trend growth, pulse amplification, step growth, and horizontal fluctuation, which correspond to the experimental test results presented later. Table 3 shows the sample distribution for each model.

The specific meanings and criteria for determining the four timing patterns of current changes in Table 3 are as follows:

- Trend growth: The current intensity shows a linear or power-law growth trend with irradiation duration; that is, the current data fluctuates randomly around a certain linear or power-law increasing function.
- Pulse amplification: The current intensity fluctuates approximately steadily and randomly with irradiation duration, and at one or more moments, a pulse response far greater than the horizontal value occurs (single point abnormal fluctuation).
- Step growth: The current intensity fluctuates approximately steadily and randomly with irradiation duration. At a certain moment, a pulse response far greater than the horizontal value occurs, and then it falls back and locks at a bigger level value, approximately fluctuating steadily and randomly. The current variation in this “amplification–fallback–locking” mode may occur multiple times during the irradiation process of the same sample. There is a current change sample with an “amplify–lock–re amplify–lock” mode.
- Horizontal fluctuation: The current intensity fluctuates randomly around the horizontal value with irradiation duration, and the fluctuation amplitude varies at different time periods. There are a large number of small outliers in stable random fluctuations.

As shown in Table 3, the sensitivity of the drain and gate to different modes varies. The drain has a greater impact on trend growth, while the gate has a greater impact on horizontal fluctuations and mixed form. The reason for this statistical result may be related to the extraction of electrons by the drain and the quality of the gate. The changes in lattice structure after irradiation are mainly caused by thermal effects, which mainly affect the gradual changes in current. Due to the relatively large drain voltage, the drain region has a strong attraction to electrons. With the continuation of irradiation, the drain region often forms excessive electron accumulation. The enhancement of the thermal effect caused by the accumulation and collision of free electrons is a gradual process, and so is the damage to the lattice structure. The trend increase in drain current is a direct representation of this change in lattice structure.

When the gate has a strong ability to control the internal electric field of the device, it reflects that the gate dielectric has better robustness. Therefore, the gate current is mostly shown as horizontal fluctuations. The mixed form refers to the horizontal fluctuations of the current level in the early stage of irradiation and the change in pulse characteristics or trends in the middle and late stages. The possible reasons for this phenomenon are as follows: (1) There are defects in the quality of the gate of the device; (2) The incident events pass directly through the gate region to form a current microchannel. The above statistical conclusions are consistent with the experimental results; that is, in most of the experimental results, the device often experiences a single-event burnout effect rather than a single-event gate rupture effect.

(2) The correlation between gate and drain current

The synchronization and statistical correlation of gate and drain current morphology changes are evaluated based on data scatter plots and simple correlation coefficients. Data analysis categorizes samples into three main types:

- Strong correlation: The gate and drain currents are significantly correlated and can be fitted and described by power or linear functions, with a sample proportion of 29%.
- Weak correlation: The statistics of gate and drain currents are not correlated, but the data form has obvious temporal synchronization, with a sample proportion of about 31%.
- No correlation: There is no statistical correlation or temporal synchronization between gate and drain currents, and the sample proportion is about 40%.

In the sample class with long irradiation duration, the gate and drain current are often strongly correlated, or the gate current level fluctuates while the drain current tends to increase.

In the sample class of short irradiation duration, the gate and drain current mostly show a weak correlation of synchronous pulse amplification.

In the sample class of general irradiation duration, the data forms of gate and drain currents have diversity.

## 3. Data Analysis

### 3.1. Data Transformation

In this paper, the experimental data are analyzed in depth, and the microscopic carrier number characteristics and irradiation effect mechanism inside the device are interpreted through the macroscopic gate and drain current intensity data in reverse. Therefore, the following data transformation methods are proposed.

If the current intensity at time *t* is *I*(*t*), then d*I*(*t*)/d*t* not only expresses the instantaneous fluctuation of current intensity *I* but also maps to the dynamic changes in the transport of carriers inside the device and the accumulation in the adjacent area of the electrode under the action of the electric field force. The discretization of d*I*(*t*)/d*t* approximation is based on a sampling step of 0.01 s for experimental data over time.

(1) $$\Delta I=I_{t+1}-I_{t},\;t=1,2,\cdots ,n$$

Further transformation to eliminate stationary noise caused by no radiative factors in differential data so that,

(2) $$I_{FC}=\left\{\begin{array}{c} \Delta I+\varepsilon ,\;\Delta I<-\varepsilon \\ 0,\;-\varepsilon \leq \Delta I\leq \varepsilon \\ \Delta I-\varepsilon ,\;\varepsilon <\Delta I \end{array}\right.$$

In the application, ∆*I* is calculated from the current data after irradiation, *ε* = 0.75 R > 0, where *R* is the range of the corresponding current data before irradiation.

For simplicity, it may be called *I*_FC_ as the fluctuation–collapse (FC) transformation of current intensity *I*, and *ε* is the collapse parameter.

### 3.2. CC Mapping Theory

#### 3.2.1. Instantaneous CC Mapping

- The significance of drain-*I*_FC_ transformation

**Figure 1:**
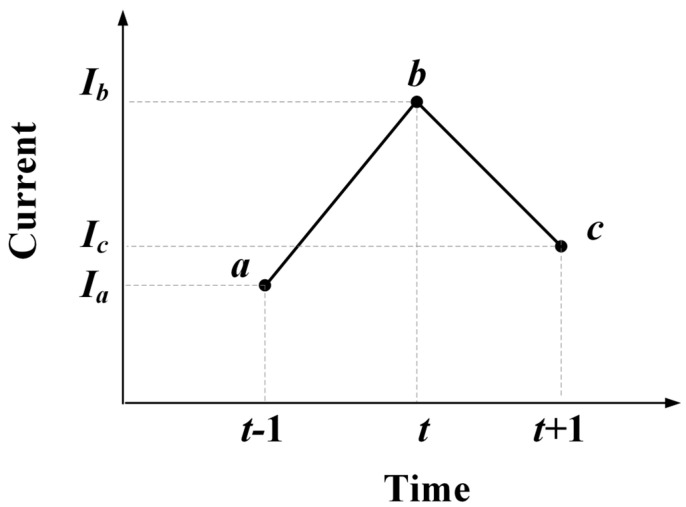
Schematic diagram of changes in current intensity.

Figure 1 shows a schematic of the changes in the current intensity at three adjacent time points.

In Figure 1, assuming that the current intensity increases from *I_a_* to *I_b_* per unit time from time *t* − 1 to time *t*, with an *I*_FC_ > 0, it indicates that at time *t* − 1, the carrier generation rate excited by heavy-ion incident excitation is greater than the recombination rate, and the electron concentration inside the ohmic contact interface increases, leading to an increase in the number of electrons that can be extracted from the drain, increasing the drain current.

The current intensity decreases from *I_b_* to *I_c_* per unit time from time *t* to time *t* + 1, with *I*_FC_ < 0, indicating that there is no new heavy ion incident at time *t.* Because of carrier recombination and electrode extraction of electrons within the previous unit time, secondary excitation of carriers is suppressed, resulting in a decrease in electron concentration inside the ohmic contact interface and a decrease in the number of extractable electrons at the drain, leading to a decrease in drain current.

*I*_FC_ = 0 indicates that the electron concentration inside the ohmic contact interface maintains dynamic equilibrium, and the fluctuation of current per unit time is white noise.

b. The significance of gate-*I*_FC_ transformation

After the heavy ion incident, the hole accumulation at the gate interface makes the induced electric field of the gate medium enhanced, resulting in the weakening of the insulating properties of the gate medium, and under the influence of this electric field, the electron percolation of the gate medium is enhanced, which further increases the number of excited electrons inside the gate medium, widens the electron channel, and the gate current rises.

#### 3.2.2. Full-Time CC Mapping Analysis

If the drain current increases continuously for two units of time from time *t* − 1 to time *t* + 1, i.e., *I_a_* < *I_b_* < *I_c_*, the influencing factors include: ① Continuous low-kinetic-energy heavy-ion incidence; ② At *t* − 1 moment, the carrier generation rate is greater than the recombination rate after the heavy ion incident, and the electron accumulation exceeds the electrode extraction capacity, increasing the internal entropy energy of the device; ③ Before the *t* − 1 moment, the sustained secondary excitation effect of the high-kinetic-energy heavy-ion incident causes the internal entropy energy of the device to continue to increase.

If the current decreases continuously for two units of time from moment *t* − 1 to moment *t* + 1, that is, *I_a_* > *I_b_* > *I_c_*, it indicates that there were no new heavy ions incident during this period, the carrier recombination rate was greater than the generation rate, the collision ionization process weakened, and the electron concentration inside the ohmic contact interface continued to decrease. There is a possibility of restructuring the electric field distribution.

The meaning of CC mapping for two consecutive units of time can be extended to the principle of CC mapping analysis over the entire irradiation time domain. Therefore, we define statistical measures.

(3) $$\mathrm{CR}=\frac{Number\;of\;data\;points\;for\;I_{FC}>0}{N}\times 100\%$$

(4) $$\mathrm{ER}=\frac{Number\;of\;data\;points\;for\;I_{FC}<0}{N}\times 100\%$$

(5) EqR=1−CR−ER

where *N* is equal to the total time points of experimental data −1.

Assuming $\forall t\in T,I_{t\text{-}drain}\geq 0$, the statistic CR represents the proportion of time that the irradiation current is enhanced throughout the entire process, mapping the accumulation characteristics of electron concentration in the region adjacent to the drain electrode; ER represents the proportion of time that the irradiation current falls back throughout the entire process, mapping the characteristics of electron concentration reduction and the reconstruction of the state distribution of internal physical quantities in the device; EqR represents the proportion of time that the current level fluctuates throughout the irradiation process, including two scenarios: stable initial current fluctuations and high-level current fluctuations caused by heavy-ion incident disturbances. It maps the characteristics of a constant electron concentration or stage invariance, which, to some extent, reflects the radiation resistance of the device.

Let CE = CR/ER, which is referred to as the carrier dynamic equilibrium ratio. CE ≈ 1 indicates a dynamic equilibrium of the rise and fall of the drain current until irradiation interruption, mapping the dynamic equilibrium of the electron concentration in the region adjacent to the drain electrode, resulting in a lower risk of electron accumulation. CE >> 1 indicates that until irradiation interruption, the drain current has a stronger upward trend than a downward trend, and there are excess free electrons in the area adjacent to the drain electrode, forming entropy energy accumulation and posing a higher risk of electric heating effect. In contrast, the electric heating effect caused by entropy energy accumulation in the region adjacent to the CE << 1 mapping drain is relatively gentle.

If $\forall t\in T,I_{t\text{-}gate}\leq 0$, the direction of current enhancement is opposite to the indicated direction, and it is necessary to flip the meaning of cognitive CR and ER, it is possible to set CE = ER/CR to maintain the invariance of the CE statistic.

## 4. Conjectures and Evidence-Based on Data Analysis

### 4.1. Simulation Evidence

In this paper, a single-event effect simulation study is carried out on a classical 150 V rated N-channel VDMOS structure to analyze the effects of factors such as incident position and bias conditions on the distribution characteristics of important physical quantities such as electron and hole concentration distributions, impact-gen rate distributions, electric field strength distributions, lattice temperature distributions, and so on.

Based on the current simulation results, six representative time points are selected, namely (1) before ion incidence; (2) 4 ps after ion incidence; (3) 0.1 ns after ion incidence; (4) 1 ns after ion incidence; (5) 10 ns after ion incidence; and (6) 100 ns after ion incidence.

**Figure 2:**
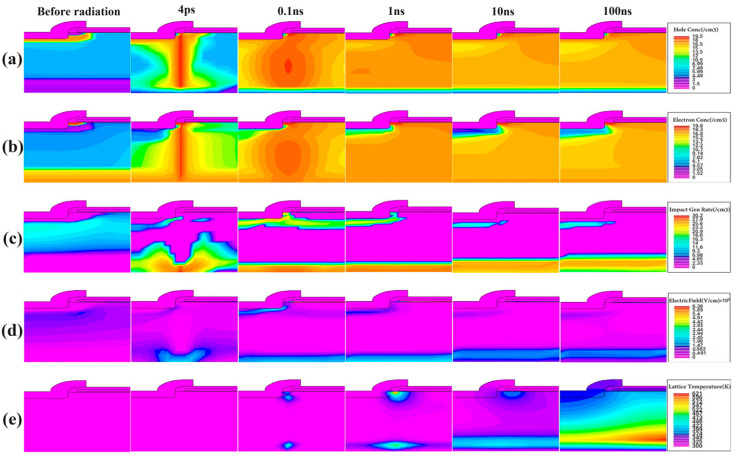
The single-event effect of 150 V VDMOS (*V*_gs_ = 0 V, *V*_ds_ = 40 V, heavy ions incident directly above the channel). (**a**) Hole concentration; (**b**) Electron concentration; (**c**) Impact gen rate; (**d**) Electric field; (**e**) Lattice temperature.

Figure 2 shows six-time-point screenshots of the simulation results of the main physical quantities of heavy ions incident directly above the channel under bias conditions *V*_gs_ = 0 V, *V*_ds_ = 40 V. Simulation results show that when the SEB effect occurs in the device, the distribution range and value of each physical quantity inside the device tend to increase.

In Figure 2a,b, before irradiation, due to the influence of doping and bias conditions, the concentration distribution of holes and electrons is different. The high-concentration region of holes is in the P-well region, and the high-concentration region of electrons is in the N^+^ source and substrate adjacent to the drain. After irradiation, PN junctions with different states such as N^+^P^−^, P^−^N^−^, and N^−^N^+^ are distributed on the track of heavy ion incidence, which, combined with the kinetic energy of the incident heavy ions, form a columnar high-concentration region. Due to the formation of electron–hole pairs during collisional ionization, the evolution of hole concentration distribution is similar to that of electron concentration distribution, with the difference being the superposition effect of distribution morphology under the influence of the pre-irradiation bias electric field. Due to the carrier extraction effect of the electrode, isolation layers of electrons and holes are formed in the adjacent regions of the source and drain electrodes.

In Figure 2c, before irradiation, the collision ionization inside the device mainly occurs in the drift region below the P-well region. After irradiation, heavy ions are incident to “tear” the high-concentration hole distribution layer below the source and channel, impacting the original high ionization rate distribution layer. This triggers an “electron explosion” on the high-concentration electron distribution layer adjacent to the substrate at the drain, forming separate collision ionization centers at both ends of the incident track. Carrier recombination occurs almost synchronously with collisional ionization, with the initial recombination center being a narrow columnar region on the incident track, which gradually contracts to the channel over time and the recombination rate decreases. On the surface side, due to the conduction of parasitic transistors in the early stage, the ionization intensity increases and forms a high ionization rate distribution layer in the X-axis direction in the drift region. Over time, due to carrier recombination and source extraction, the ionization rate gradually decreases and contracts downwards towards the P-well region. On the drain side, the ionization intensity continues over time and gradually evolves into a band-like region with clear boundaries in the X-axis direction under the action of electrode extraction. The ionization rate becomes uniform, and the distribution area gradually expands.

The electric field intensity distribution in Figure 2d characterizes the process of carrier generation recombination and the extraction of carriers by the source and drain electrodes. Compared with the high-intensity region of the electric field located below the channel in the P-well region before irradiation, the evolution characteristics of the electric field distribution after irradiation are similar to those of the collision ionization rate. Due to the accumulation of a large number of holes generated after irradiation, the field strength of the gate oxide layer is significantly higher than that of other regions. At the same time, the accumulation of high-concentration electrons leads to the formation of a relatively high field-strength distribution layer near the substrate at the drain.

The distribution characteristics and evolution law of lattice temperature in Figure 2e are highly correlated with (c) and (d), and the thermally sensitive areas inside the device correspond to the high concentration areas of collisional ionization. The early lattice temperature increases slowly with irradiation duration. In the later stage, the thermosensitive region of the substrate near the drain rapidly expands, and the lattice temperature of the gate oxide layer and the substrate near the drain increases nonlinearly, indicating an explosive growth in the accumulation of disordered charge carriers in the corresponding region. When the lattice temperature exceeds the threshold value of the material’s stable operating temperature, irreversible damage to the device will be triggered.

On this basis, under the condition of *V*_gs_ = 0 V, we comparatively analyze the distribution characteristics and evolution laws of the physical quantities after heavy-ion irradiation at *V*_ds_ = 30 V, 40 V, and 50 V. The simulation results show that the distribution of each physical quantity at *V*_ds_ = 30 V is similar to that at *V*_ds_ = 40 V, with a small peak, and then gradually converges with that before irradiation as the distribution of each physical quantity is reconstructed in the middle of the irradiation duration, and the distribution of each physical quantity at *V*_ds_ = 50 V is basically the same as that at *V*_ds_ = 40 V, with a large peak.

In this paper, we also investigate the changes in the key parameters after the heavy ions are incident at different locations on the device surface under the bias conditions of *V*_gs_ = 0 V and *V*_ds_ = 40 V. Compared with incident directly above the channel, the response characteristics and evolution patterns of the key physical parameters inside the device are similar when heavy ions are incident directly above the source electrode, with slightly higher values of collisional ionization rate and electric field strength in the region immediately adjacent to the source electrode, but slightly lower values of carrier concentration and lattice temperature, and a relatively lower leakage current level and peak value. After heavy ion incidence directly above the gate, the carrier complexation rate near the drain electrode is higher, the entropy energy accumulation level of the substrate is low, the thermal stresses mainly act in the region immediately adjacent to the gate electrode, and the leakage current and lattice temperature of the substrate are much lower than those at the other two locations.

**Figure 3:**
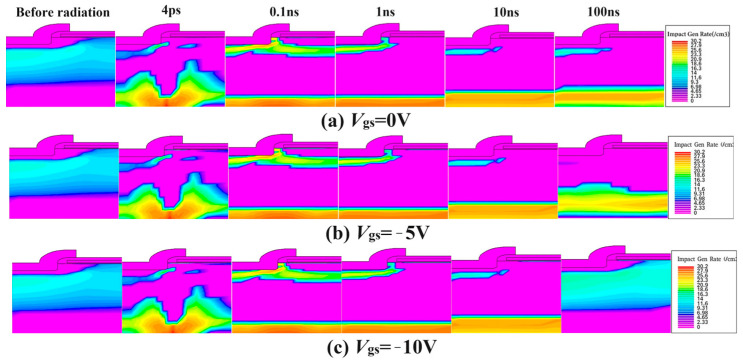
Distribution of impact gen rate under different gate bias conditions ($V_{ds}=40\;V$).

As for the influence of gate bias on the internal physical quantity of the device under the condition of ion incidence, we focus on the analysis of the influence of different gate voltages on the carrier impact ionization rate of the device, as shown in Figure 3.

Under the same drain voltage, in the first half of the collision ionization process after heavy ion incidence, under gate bias *V*_gs_ = 0, −5, −10 V conditions, the distribution characteristics of various physical quantities are almost the same. As the collision ionization process spreads, the electric field reconstructs, and the gate bias effect becomes apparent. As the gate bias voltage increased, the collision ionization center moved towards the surface, the entropy energy of the drain electrode adjacent to the substrate weakened, the drain current decreased, the thermal stress of the gate oxide layer increased, and the risk of radiation damage events increased.

### 4.2. Single-Event Effect CC Mapping Analysis

In this section, based on the CC mapping analysis results of the single-event effect for all 55 samples, six samples are preferred for detailed elaboration in combination with the current timing patterns mentioned in Part 2. The gate/drain voltage drift has a significant influence on the irradiation interruption mode. Under stable gate bias, the drain voltage drift is inversely proportional to the irradiation delay. Severe drain voltage drift tends to trigger drain overcurrent interruption earlier. Gate bias instability is the main factor that triggers gate overcurrent interruption. Table 4 lists the main parameters of CC mapping analysis for the case samples.

Table 5 lists the collapse parameters of the gate and drain current FC transformations based on the case sample bias data.

The examination of all 55 sample bias data and their FC transformation data supports the theoretical hypotheses in the previous section.

#### 4.2.1. Single-Event Effect of Sample VII-1

**Figure 4:**
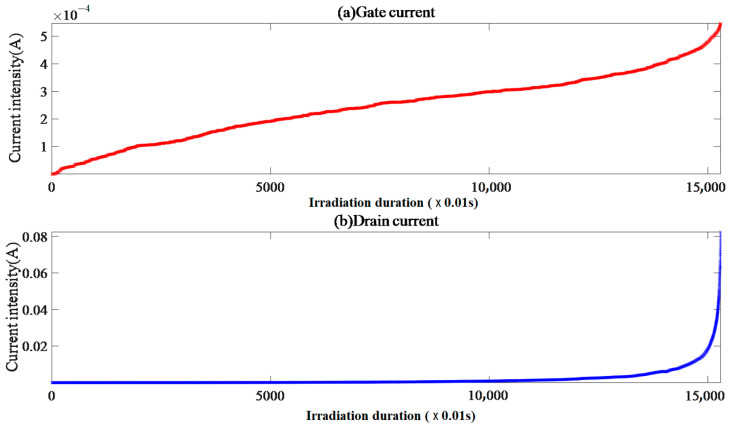
Sample VII-1 gate and drain current image.

Sample VII-1 has an initial gate and drain current of 4.04 × 10^−11^ A and 4.59 × 10^−9^ A, respectively, shown in Figure 4. After supplying the beam, the gate current shows an S-shaped nonlinear trend of increasing, and the drain current shows a power-law trend of increasing. There is a strong correlation between the gate and drain currents, and the “gate–drain current curve” can be fitted by a power function. The drain-triggered overcurrent interruption.

The gate and drain current FC data take FC = 0 as the peak point; the gate current is approximately symmetrically distributed, and the drain current has a positively skewed peak distribution.

The synchronous growth trend of the gate and drain current after the beam supply indicates that heavy ion incidence has a direct impact on the number of charge carriers in the region adjacent to the gate and drain, inferring the existence of two collision ionization centers near the surface and substrate. Combined with the current image analysis, the ratio of the gate to drain current EqR values is about 0.36, indicating that the initial gate response is more sensitive after heavy ion incidence. The gate current CE = ER/CR ≈ 1.10 indicates that with irradiation duration, the insulation performance of the gate oxide layer continues to deteriorate, and there is electron flow into the region adjacent to the gate. After carrier recombination, a small amount of electrons flow into the drain, but the electric field control function of the gate is not completely ineffective. The drain bias rate *V*_ds_/*BV* ≈ 0.33 and drain current CE = CR/ER ≈ 3.20 indicate that the drain has a relatively high attraction to electrons. With the irradiation duration and decrease in the gate’s ability to regulate the electric field, the continuous electron influx at the source and gate and the ionization of the electrothermal effect are enhanced. A large amount of electron accumulation is generated in the region adjacent to the drain, and the entropy energy explodes before irradiation interruption, leading to irreversible damage to the substrate lattice structure, that is, the occurrence of SEB events.

#### 4.2.2. Single-Event Effect of Sample VII-5

**Figure 5:**
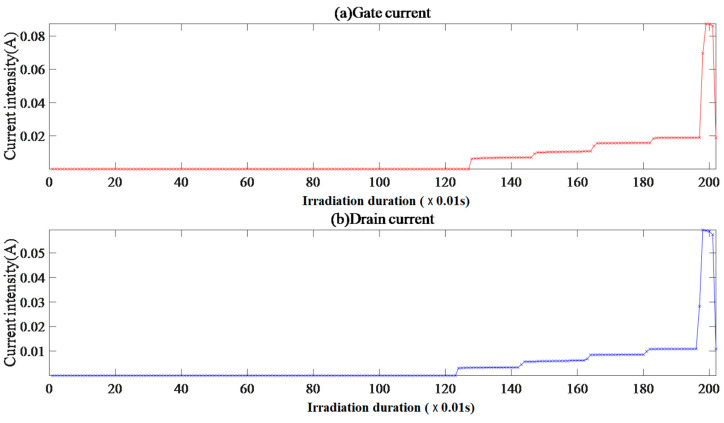
Sample VII-5 gate and drain current image.

Sample VII-5 has an initial gate current of 1.99 × 10^−10^ A and a drain current of 4.62 × 10^−9^ A, with a strong correlation between the gate and drain currents, which is shown in Figure 5. The result presents a synchronous stepwise growth data form, after which both the gate and drain voltages fluctuate steadily and randomly, with a duration of 1.01 s. The gate and drain voltage drifted synchronously, followed by a step amplification of the drain current slightly before the gate current and a duration of 2.02 s when the gate triggered an overcurrent interruption before the drain.

Note that the captured gate transient peak current of 101.6 mA is not displayed in the figure. The gate and drain current FC data were approximately distributed discretely, and the probability of other points was relatively small, except for FC = 0.

Because *V*_gs_ = −10 V, the internal electric field of the device is formed by the superposition of the source-drain longitudinal electric field and the gate-source transverse electric field. The gate oxide layer is a key structure for electric field regulation, and the difference in the thermal expansion coefficients between materials leads to a certain degree of electron mobility in the gate oxide layer under the electrothermal effect.

The synchronous drift of the gate and drain voltages with a duration of 1.01 s after the beam supply indicates that the internal electric field of the device is affected by heavy-ion incidence. After a duration of 1.23 s and 1.27 s, the drain and gate currents undergo stepwise amplification one after another. It is not difficult to understand that the period from the incidence of heavy ions to the amplification of the drain current should be the process of collision ionization to produce charge carriers.

Until the irradiation interruption, the EqR values of the drain and gate currents were 59.20 and 62.19, respectively, indicating that the time proportion of the single-event effect process was 40.8% on the drain side and 37.81% on the gate side, indicating that the single-event effect on the drain side diffused to the gate side. The drain current CE ≈ 7.17 indicates that the irradiation effect forms electron accumulation in the region adjacent to the drain, and the enhanced entropy energy affects the physical quantities and their distribution, such as the internal electric field strength and lattice temperature of the device. The gate current CE ≈ 11.65 indicates that the irradiation effect causes electrons to enter the drift region through the gate oxide layer and participate in carrier recombination in the drift region. Due to the electric heating effect of the gate oxide layer, the electric field control function of the gate is damaged, and excess electrons flowing from the gate are attracted by the drain under the action of the electric field force and accumulate on the substrate.

The stepwise amplification phenomenon of the current reflects the electrothermal effect of the excess electron increment on the substrate, which further damages the electric field control function of the gate oxide layer. The transient stability of the stepwise current amplification reflects the recombination effect of the carriers and the response time of the lattice temperature. The drain and gate current pulse amplification before irradiation interruption is a critical manifestation of radiation hard damage events in devices, and the gate overcurrent interruption indicates irreversible failure of the gate oxide layer.

#### 4.2.3. Single-Event Effect of Sample I-4

**Figure 6:**
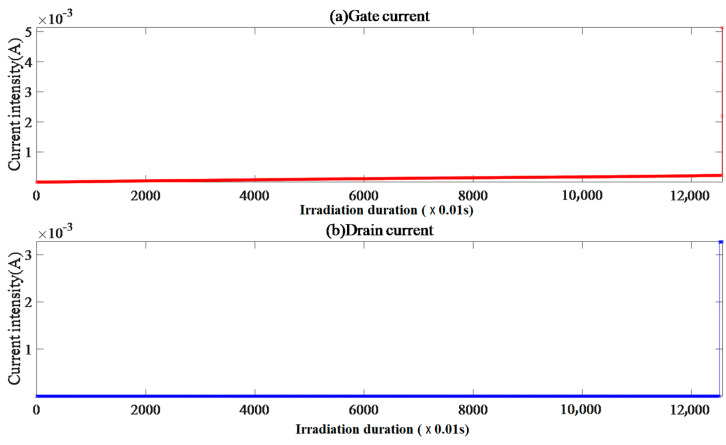
Sample I-4 gate and drain current image.

Sample I-4 exhibited a weak correlation with the drain current, which is shown in Figure 6. The gate current shows a linearly increasing trend, while the drain current fluctuates steadily and randomly. The synchronous pulse amplification had a duration of 125.5 s and no overcurrent interruption was triggered. The peak currents were approximately equal, and the gate current was slightly greater than the drain current.

The gate and drain current FC data had a peak point of FC = 0, with a slightly negatively skewed peak distribution of the gate current and a symmetrical peak distribution of the drain current.

Combining the current image analysis. On the gate side, EqR = 9.10, indicating that the duration of stable and random fluctuations in the gate current after a heavy ion incident is extremely short, and a small linear increase in current indicates electron permeation in the gate oxide layer. The gate current CE ≈ 1.08, indicating that the electron generation rate in the region adjacent to the gate is greater than the recombination rate. On the drain side, the linear growth rate of the drain current is much lower than that of the gate current. EqR = 72.16 indicates that the influence of the early current fluctuations on the internal electric field strength of the device can be ignored. CE ≈ 1.03, indicating that electrons slowly accumulate in the area adjacent to the drain. In the later stage of irradiation, the entropy energy of the substrate increases, and ionization intensifies. The thermal stress of the substrate lattice causes a sudden valence bond fracture before irradiation interruption, and the drain current is amplified by the pulse first. The holes generated by substrate ionization are attracted by the gate, which further enhances the electric field strength of the gate oxide layer. The amplification of the gate current pulses is a duration response to substrate ionization, which causes a sharp increase in the number of electrons flowing into the gate oxide layer.

In the experiment, the total fluence amount of sample I-4 exceeded the threshold, and irradiation was interrupted. However, the dual-pulse response of the gate and drain current before irradiation interruption indicated that the device was in a critical state of irradiation hard damage. Based on the prediction of the pulse current intensity, the probability of SEGR events occurring in the device was slightly higher than that of the SEB.

#### 4.2.4. Single-Event Effect of Sample VIII-1

**Figure 7:**
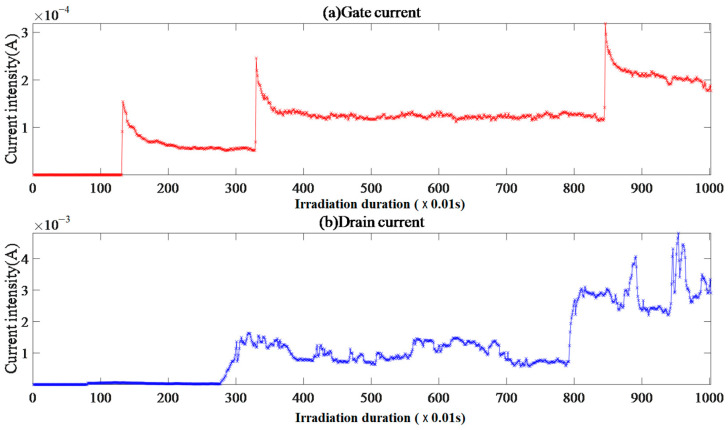
Sample VIII-1 gate and drain current image.

In Figure 7, sample VIII-1 showed a weak correlation between the gate and drain currents, with a typical step potential increase in the gate current and a fusion of drain current trends and horizontal fluctuations. The drain triggered an overcurrent interruption.

The gate and drain current FC data take FC = 0 as the peak point without considering outliers. The gate current had a positively skewed peak distribution, whereas the drain current had a symmetrical peak distribution. The FC outlier corresponded to the current pulse amplification (step) point.

On the gate side, the stepwise increase in the gate current exhibits three significant pulse responses, reflecting three large kinetic energy heavy-ion incidence and collision ionization processes. The current step increase here is different from that of Sample VII-5. In general, changes in the physical quantities inside the device are continuous. Based on this theoretical understanding, the small-step characteristics of the sample VII-5 current are understood as the duration response of the carrier generation recombination and electrothermal effects. However, the step characteristics of the gate current in sample VIII-1 represent abnormal disturbances, which can be understood as repeated occurrences of heavy ions at unequal intervals. Pulse amplification indicates an abnormal transient influx of a large number of electrons into the gate, and the subsequent recombination process, to some extent, suppresses the continued rise of the electrothermal effect. EqR = 15.09 characterizes the stability of the gate current in the initial stage and after each pulse drop. CE = 0.87, indicating that the carrier recombination process in the region adjacent to the gate is relatively active, which is the basic condition for maintaining high current locking. Note that the step-by-step increase in the gate current indicates that the entropy energy in the region adjacent to the gate also increases step by step. The thermal stress effect causes the gate function to continue to degrade, thereby affecting the physical quantity evolution process of the substrate.

On the drain side, EqR = 8.30 and CE ≈ 0.98, indicating that the dynamic balance of the drain carrier is better than that of the gate, but the carrier transport intensity near the drain is higher. From Figure 7, the amplification of the gate current strong pulse occurs before the drain current. It can be understood that the trend growth process of the drain current is influenced by the electric heating effect on the gate side.

The irradiation process was interrupted owing to drain overcurrent, resulting in damage to the SEB of sample VIII-1. The most likely reason for this result is the direct impact of high-energy events in the neck region. Because *V*_gs_ = 0 V and *V*_ds_ = 150 V, under the action of an extremely high drain potential, some of the electrons flowing in from the gate combine with the surface holes, and another part enters the substrate for accumulation. As the gate current increases, the gate field control ability decreases, and the difference in heat resistance between the Si and SiO_2_ materials leads to lattice structure disorder of the substrate, resulting in an instantaneous large current drain from the drain.

#### 4.2.5. Single-Event Effect of Sample II-1

**Figure 8:**
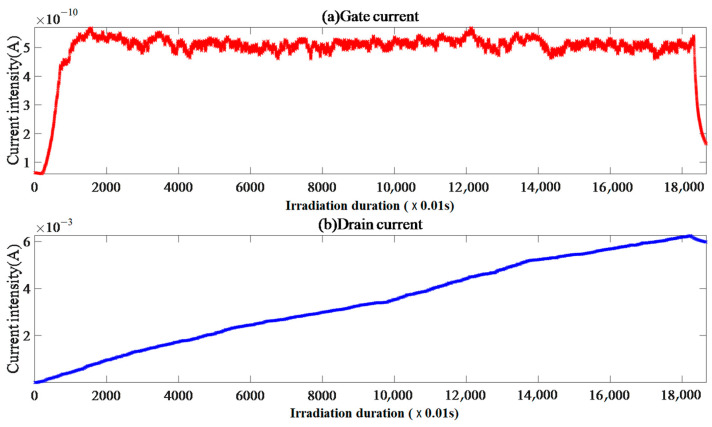
Sample II-1 gate and drain current image.

There was no correlation between the sample II-1 gate and the drain current shown in Figure 8. The gate current rapidly drifts from the initial current to approximately horizontal fluctuations after 5 × 10^−10^ A. The approximate linear trend of the drain current increases, and the total fluence exceeds the limit and is interrupted. It is predicted that the drain electrode will trigger overcurrent interruptions with high probability.

The gate current FC data have a positively skewed peak distribution with FC = 0 as the peak point and a negatively skewed distribution with low drain current peaks.

The gate current increases approximately linearly from 1.27 s to 16 s after beam supply, indicating a transition from electron permeation to thermal breakdown within the gate oxide layer. The electron channel expands the current intensity to 5 × 10^−10^ A, then switches to a heteroscedasticity fluctuation state until the drain current drops from the peak value of 9.6 in 180 s × 10^−3^ A. This drop causes a synchronous drop in the gate current, resulting in a total fluence exceeding the limit and interruption. The high gate current EqR and gate CE ≈ 0.82 reflect the effective field control function of the gate during irradiation and the relatively low entropy energy accumulation of the gate oxide layer. The drain current EqR was small, indicating that the drain was immediately affected by heavy ion incidence after the beam was supplied. The drain CE ≈ 0.62 indicates that the electrons accumulated in the drain adjacent to the substrate could be effectively extracted. The linear increase in the drain current reflects an increase in the total fluence, and the drain current drops from its peak before the total fluence exceeds the limit, indicating that the electric heating effect generated by the accumulation of device entropy energy is lower than the tolerance threshold of the material at a larger fluence.

Therefore, it is speculated that the incident position of heavy ions avoids collision ionization-sensitive positions, such as the gate oxide layer, channel, and neck region, and the incident kinetic energy of heavy ions is relatively small. This is a typical example of reversible radiation damage.

#### 4.2.6. Single-Event Effect of Sample IV-2

**Figure 9:**
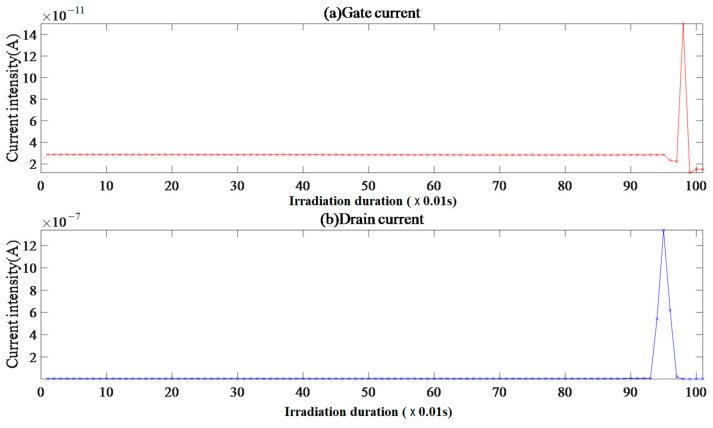
Sample IV-2 gate and drain current image.

In Figure 9, sample IV-2 shows a weak correlation between the gate and drain currents, with stable and random fluctuations after the beam supply. The drain current is amplified twice in a row with a duration of about 0.94 s, followed by gate current pulse amplification, which is delayed to 1.01 s and triggers overcurrent interruption by the drain.

Note that the captured transient peak current of 5.77 A is not displayed in the figure. The gate current FC data were distributed discretely, and the probability of points other than FC = 0 could be ignored.

Because *V*_gs_ = 0 V and *V*_ds_ = 150 V, the voltage drift caused by the overcurrent interruption from the beam to the drain can be ignored. The extremely high gate current EqR value and CE = 1 indicate that there is almost no electric heating effect on the gate side of sample IV-2 during irradiation. The extremely high drain current EqR value and CE ≈ 1.67 indicate that after the beam is supplied, heavy ions directly enter from collision ionization-sensitive positions, such as channels, and undergo intense collision ionization near the drain electrode substrate, forming electron accumulation and instantaneous drain from the drain current, leading to the occurrence of SEB events.

## 5. Conclusions

This article is based on the classification and recognition of the current characteristics and temporal morphology after heavy-ion irradiation. Using simulation methods, the internal carrier distribution and physical field evolution characteristics of the device are discussed, and a preliminary CC mapping analysis paradigm for single-event irradiation experimental data of Si MOSFET devices is established. Summarizing the discussion in this article, the following conclusions can be drawn: CC mapping analysis is a feasible evaluation method for single-event effects. The difference collapse transformation of the experimental data proposed in this study, as well as the three statistical variables CR, ER, and EqR, can better characterize the macroscopic characteristics of current fluctuations in the irradiated sample device. By observing the simulation results in collaboration, it is possible to understand the distribution and evolution characteristics of the device carriers from the current temporal morphology of the experimental data. It helps evaluate the single-event effects and damage properties of the devices.

## Figures and Tables

**Table 1:** Sample distribution of different device types.

Type	I	II	III	IV	V	VI	VII	VIII
Number	8	10	7	3	7	7	6	7
Gate Structure	planar	split-gate	planar	split-gate	planar	split-gate	planar	planar
Channel	N	N	N	N	N	N	N	N

**Table 2:** Two-factor sample distribution law of interrupt mode and irradiation duration (%).

Interrupt Method	Irradiation Duration			Total
	0 s ≤ *t* < 10 s	10 s ≤ *t* < 100 s	*t* ≥ 100 s	
Drain overcurrent	15	22	19	56
Gate overcurrent	3.5	3.5	-	7
Fluence overlimit	-	-	37	37
**Total**	18.5	25.5	56	100

**Table 3:** Sample distribution law of current data pattern type (%).

Electrode	Time Series Morphology of Current Intensity				
	Trend Growth	Pulse Amplification	Step Growth	Horizontal Fluctuation	Mixed Form
Gate	28	9	28	19	16
Drain	57	10	26	2	5

**Table 4:** Sample distribution law of current data form types. Case sample CC mapping analysis main parameters.

*NO.*	*BV*	Bias Voltage and Irradiation Stability				Radiation Results		
		*V*_GS_	*V*_DS_	*V*_GS-DRIFT_	*V*_DS-DRIFT_	Interrupt Method	Irradiation Duration	Current Peak
VII-1	1500 V	0 V	500 V	3 V	−4.8 V	Drain overcurrent	152.90 s	100.4 mA
VII-5	1500 V	−10 V	450 V	1.8 V	4.0 V	Gate overcurrent	2.02 s	101.6 mA
I-4	650 V	−5 V	150 V	1.2 V	−4.0 V	Fluence overlimit	125.69 s	5.2 mA(gate)
VIII-1	650 V	0 V	150 V	1.0 V	−3.9 V	Drain overcurrent	10.00 s	2.39 A
II-1	150 V	0 V	50 V	0.1 V	−4.9 V	Fluence overlimit	186.73 s	9.6 mA(gate)
IV-2	40 V	0 V	30 V	0 V	0.8 V	Drain overcurrent	1.01 s	5.77 A

**Table 5:** Case sample FC transformation collapse parameter.

Number	VII-1	VII-5	I-4	VIII-1	II-1	IV-2
∆_GS_	$7.19\times 10^{-14}$	$7.22\times 10^{-13}$	$4.37\times 10^{-13}$	$8.79\times 10^{-8}$	$1.34\times 10^{-13}$	$6.34\times 10^{-14}$
∆_DS_	$1.77\times 10^{-11}$	$1.61\times 10^{-11}$	$1.50\times 10^{-8}$	$6.58\times 10^{-8}$	$1.50\times 10^{-11}$	$1.58\times 10^{-9}$

## Data Availability

The original contributions presented in the study are included in the article, further inquiries can be directed to the corresponding author.
